# Live Birth Rate of Frozen-Thawed Single Blastocyst Transfer After 6 or 7 Days of Progesterone Administration in Hormone Replacement Therapy Cycles: A Propensity Score-Matched Cohort Study

**DOI:** 10.3389/fendo.2021.706427

**Published:** 2021-08-11

**Authors:** Xinhong Yang, Zhiqin Bu, Linli Hu

**Affiliations:** Reproductive Medical Center, Henan Province Key Laboratory for Reproduction and Genetics, The First Affiliated Hospital of Zhengzhou University, Zhengzhou, China

**Keywords:** frozen-thawed embryo transfer, hormone replacement treatment, live birth rate, progesterone administration, blastocyst transfer

## Abstract

**Background:**

Progesterone administration before transfer in hormone replacement treatment (HRT) is crucial to pregnancy outcomes in frozen-thawed blastocyst transfer (FET), but the optimal progesterone duration is inconsistent. The objective of this study was to investigate live birth rate (LBR) of different progesterone duration before blastocyst transfer in HRT–FET cycles.

**Method:**

In this retrospective cohort study, patients underwent first HRT–FET (including suppression HRT) from January 2016 to December 2019 were included. Logit-transformed propensity score matching (PSM) was performed to assess covariates. The primary outcome was live birth rate after 28 weeks’ gestation. Basing on different duration of progesterone before transfer, patients were classified into P6-protocol (blastocyst transfer performed on the sixth day), or P7-protocol (blastocyst transfer performed on the seventh day). Subgroup analyses were conducted as follows: age stratification (–35, 35–38, 38–), development days of blastocyst (D5 or D6), blastocyst quality (high-quality or poor-quality), and endometrial preparation protocols (HRT or suppression HRT).

**Result:**

After case matching with propensity score methods, a total of 1,400 patients were included finally: 700 with P6-protocol and 700 with P7-protocol. Significantly higher live birth rate (38.43% *versus* 31.57%, respectively, P = 0.01) and clinical pregnant rate (50.43% *versus* 44.14%, respectively, P = 0.02) were observed in P6-protocol than those of P7-protocol. First-trimester abortion rates (18.13% *versus* 20.71%, P = 0.40) and ectopic pregnancy rates (2.27% *versus* 1.94%, P = 0.77) were similar between P6- and P7-groups. Preterm birth rate, low birth weight rate, newborn sex proportion, neonatal malformation rate were comparable between groups. Significantly higher LBRs were observed in patients with: age under 35, D5 blastocyst transfer, high-quality blastocyst transfer, and undergoing HRT cycles combined P6-protocol.

**Conclusion:**

Frozen-thawed blastocyst transfer on the sixth day of progesterone administration in first HRT cycle is related to higher live birth rate compared with transfer on the seventh day, especially among patients aged under 35, D5 blastocyst and/or high-quality blastocyst transfer.

## Introduction

Elective single embryo transfer policy to reduce multiple pregnancies without lowering cumulative live birth rate has become popular in assisted reproductive technology at home and abroad (1, 2). To reduce the iatrogenic risk of ovarian hyperstimulation syndrome, to perform the pre-implantation genetic testing, or to avoid embryo-endometrial asynchrony in fresh cycle, the use of ‘freeze-all’ strategy with subsequent frozen-thawed blastocyst transfer (FET) is a promising option with gratifying live birth rate and reliable safety (3–6). However, there is no consensus on the optimal endometrial preparation protocol for FET.

Various endometrial preparation protocols exist in FET cycles: true natural cycle with spontaneously ovulation, modified NC cycle with human chorionic gonadotrophin to trigger ovulation, and hormone replacement therapy (HRT) cycle without traditional ovulation (7). At present, the evidence cannot determine which scheme is best, but potentially decreased maternal and neonatal morbidity have been reported in natural cycle recently (8, 9). However, HRT cycle has been chosen widely because non-restricted by ovulation and more convenient.

Due to the absence of corpus luteum in HRT, progesterone duration before transfer is crucial to pregnancy outcomes in FET cycles (7, 10). In a natural cycle, progesterone starts to rise 2–3 days before ovulation, due to the LH-stimulated production by the peripheral granulosa cells. Endometrial receptivity could also be achieved after very short progesterone exposure, but such an approach showed a higher risk of pregnancy loss because the endometrium was insufficiently decidualized (11). Basing on available evidence, the optimal progesterone duration before transfer in HRT is assumed to be equal to the theoretical day of ovulation or 1 day later (7). In HRT cycle, blastocyst was proposed to be transferred at least on day (embryonic age + 1) of progesterone administration, annotated as “progesterone + embryonic age” (7). However, blastocysts were transferred from 5 to 7 days of progesterone administration in different HRT protocols nowadays (12, 13). Data on the optimal duration of progesterone administration before blastocyst transfer are inconsistent.

The primary objective of this study was to evaluate live birth rates between FET performed on the sixth or seventh day after progesterone administration in first single blastocyst transfer cycle during HRT treatment. The secondary objective was to investigate the effects of blastocyst development days and blastocyst quality on pregnancy outcomes.

## Materials and Methods

### Patients

This study has been approved by the Institutional Review Board (IRB) of First Affiliated Hospital of Zhengzhou University. Data in this study were from the Clinical Reproductive Medicine Management System/Electronic Medical Record Cohort Database (CCRM/EMRCD) in Reproductive Medical Center, First Affiliated Hospital of Zhengzhou University. Inclusion criteria were: [1] first single frozen-thawed blastocyst transfer cycles from January 2016 to December 2019; and [2] hormone replacement therapy (HRT), including suppression HRT. Exclusion criteria were: [1] preimplantation genetic testing cycles; [2] oocyte donation cycles; [3] uterine factors (malformation, adhesions, stages III to IV of endometriosis, ≥4 cm hysteromyoma or submucous myoma, adenomyosis); [4] systemic diseases; and [5] endometrial thickness on transfer day <7 mm. Patients’ basic parameters included maternal age, body mass index (BMI), type of infertility, endometrial thickness on the transfer day, days of embryo development, blastocyst quality.

### Endometrial Preparation and Luteal Phase Support Protocols

Endometrial preparation protocols consisted of HRT and suppression HRT. HRT cycle was applicable as described (14). HRT was applicable for patients with an irregular menstrual cycle, ovulation disorder, or poor endometrial and follicular development in NC. Starting from days 2–3 of menstruation, 2–4 mg/day of estradiol valerate (Progynova, Bayer, Germany) was administered.

Suppression HRT was applicable for patients with stages I to II of endometriosis, <4 cm hysteromyoma, endometrial growth restriction in canceled HRT cycles. On the second day of menstruation, 3.75 mg of long-acting GnRHa was used, and 2–4 mg/day of estradiol valerate (Progynova, Bayer, Germany) was administered from 28 days after GnRHa down-regulation.

Similarly, clinicians decided to maintain the original dosage or up-regulate dosage according to the thickness of endometrium. When the endometrial thickness was at least 7 mm after at least 12–14 days medication, 60 mg progesterone was additionally administered to decidualize the endometrium. According to the standard operating procedure in our center, the first doses of progesterone were given before 9 a.m. and the ET was performed about 10 a.m. on the transfer day.

Blastocyst transfer was performed on the seventh day (P7-protocol), or on sixth day (P6-protocol) after progesterone administration. Approximately 60 mg intramuscular progesterone or 900 mg progesterone sustained-release vaginal gel, in company with 20 mg oral progesterone daily, were provided from the transfer day as luteal support.

### Laboratory Protocols

Blastocysts were mainly evaluated based on three morphologic parameters: the blastocoele expansion degree, the ICM grade and the TE grade according to the Gardner and Schoolcraft’s grading system. The protocol of vitrification and warming followed the instructions established by Kuwayama et al. The specific methods were as described as published from our center (14). Vitrification was used for surplus blastocysts or cycles unsuitable for embryo transfer. Before vitrification, a 2.0-ms laser (Octax Laser Shot™ System, Germany) was used to generate a hole in the TE cell junction away from the ICM location to induce blastocoel shrinkage.

For thawing, the re-expanded blastocysts were considered suitable for transfer. Besides, laser-assisted hatching was conducted to acquire a thin zona pellucida except for hatched blastocysts.

### Definition of Outcome Measures

The primary outcome was live birth rate (LBR) after 28 weeks’ gestation. Secondary outcome was clinical pregnancy rate (CPR) defined as observation of a gestational sac inside/outside the uterine cavity *via* ultrasound. Other indicators included as follows: biochemical pregnancy rate (serum hCG testing over 50 miu/ml on 14th day after transfer), ectopic pregnancy rate (observation of a gestational sac outside uterine cavity *via* ultrasound), first trimester miscarriage (spontaneous pregnancy loss less than 12 weeks of gestation after clinical pregnant).

### Statistical Analysis

Because of non-randomized study design, we performed a matched propensity score (PSM) analysis to assess covariates (15). The PSM was estimated by multivariable logistic regression. The LBRs were analyzed by multivariable logistic regression to account for the potential confounding effect of variables known to affect live birth as previously published (13, 14). Potential con- founders were maternal age at first FET, indication for treatment, duration of infertility, BMI, endometrial preparation protocol, endometrial thickness, blastocyst development days, blastocyst quality, and progesterone administration protocol. Since none of these patients included reported a history of smoking, the association between smoking and LBR was not evaluated in the current study. Besides, progesterone duration before transfer was the independent variable in the regression model. The other parameters used in the study were also chosen as independent variables for PSM in the present study.

The logit-transformed PS matching was performed using a 1:1 ratio protocol without replacement (greedy-matching algorithm) with a caliper width of 0.1 standard deviation. Balance of covariates was judged by standardized differences. Here the balance is considered to be satisfactory when the standardized difference is less than 10% (16).

All data were included into SPSS (Statistical Package for Social Science, SPSS Inc., Chicago, IL) 26.0 for analysis. Continuous variables are presented as the mean ± SD, an categorical variables are presented as frequencies (percentages). Comparisons among different groups were performed with independent t-test, chi-square test and Fisher’s exact test. All tests were two-sided, and statistical significance was defined as P < 0.05.

## Results

### Demographics of Study Subjects

**Figure 1:**
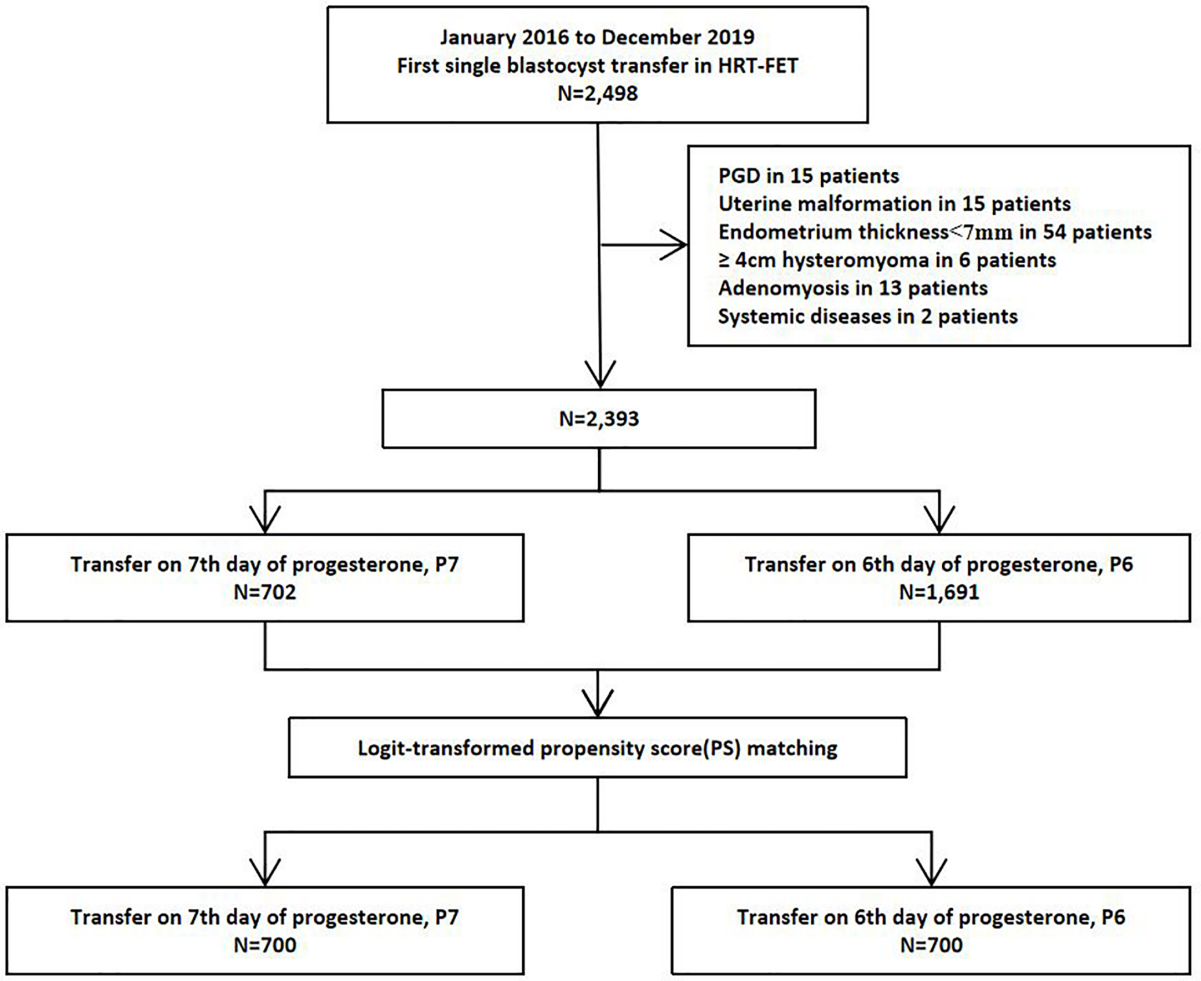
Study flow chart.

A total of 2,498 first single frozen-thawed blastocyst transfer cycles underwent HRT–FET were available during the study period. Of these, 105 cycles were excluded as shown in **Figure 1**. A total of 2,393 HRT cycles were collected from January 2016 to December 2019, including 702 cycles with P7-protocol and 1,691 cycles with P6-protocol. After logit-transformed PSM, 700 pairs were included into this study finally, with no loss of follow-up (**Figure 1**). Significantly higher live birth rate (38.43% *versus* 31.57%, *P* = 0.01) was reported in P6-protocol than that in P7-protocol respectively.

Demographic characteristics before and after PSM were presented in **Table 1**.

**Table 1:** Patient characteristics before and after propensity score matching.

	All patients (n = 2393)			PS–matched Pairs (n = 1400)		
	P7-protocol	P6-protocol	Std. Diff.	P7-protocol	P6-protocol	Std. Diff.
Parameter	n = 702	n = 1691	(%)	n = 700	n = 700	(%)
Age stratification, year						
-35, n(%)	534 (76.07%)	569 (81.05%)	-14.91	534 (76.30%)	518 (74.00%)	5.35
35-38, n(%)	83 (11.82%)	64 (9.12%)	8.04	83 (11.85%)	95 (13.61%)	5.31
38-, n(%)	85 (12.11%)	69 (9.83%)	11.55	83 (11.85%)	87 (12.39%)	1.75
Duration of infertility, year	4.24 ± 3.51	4.56 ± 3.29	-12.76	3.96 ± 3.41	3.99 ± 3.30	-0.96
BMI, kg/m^2^	23.08 ± 3.32	23.32 ± 2.94	-19.98	23.09 ± 3.32	23.13 ± 3.22	-1.21
Basal FSH, mIU/mL	6.58 ± 2.69	6.41 ± 2.10	6.17	6.53 ± 2.38	6.58 ± 2.35	-1.39
AMH, ng/ml	4.86 ± 3.89	5.07 ± 3.70	-29.40	4.25 ± 3.77	4.37 ± 3.46	-3.29
Infertility (secondary)	446 (63.53%)	467 (66.52%)	-2.54	445 (63.57%)	454 (64.86%)	-2.67
Endometrial preparation Protocol(HRT)	589 (83.90%)	595(84.76%)	-4.53	587 (83.86%)	603 (86.14%)	-6.22
Endometrium thickness, mm	9.95 ± 1.85	10.04 ± 1.78	-2.3	9.94 ± 1.84	9.90 ± 1.75	0.1
Blastocyst development days(D5)	527 (75.07%)	465 (66.24%)	18.78	525 (75.00%)	517 (73.86%)	2.64
Blastocyst quality (high-quality)	399 (56.84%)	386 (54.99%)	6.82	397 (56.71%)	371 (53.00%)	7.49

Values are presented as mean ± standard deviation or n (%).

BMI, body mass index; FSH, follicle stimulating hormone; HRT, hormone replacement treatment; AMH, anti-mullerian hormone; High-quality, both the inner cell mass and trophectoderm scores were above grade B (3BB).

Of the cohort of 2,393 patients, PS matching was possible in 700 pairs (**Table 1**). PS matching reduced the standardized differences in baseline covariates between P7- and P6- groups substantially. In the PS-matched groups, all standardized differences were <10%. The mean distance in the estimated PS was 0.03 and resulted in well-matched patients with similar baseline characteristics.

### Overall Results

Maternal pregnancy and neonatal outcomes in the overall population are listed in **Table 2**. P6-protocol was supposed to be related to better outcomes.

**Table 2:** Pregnant outcomes in overall PS-matched population.

Variable	P7-protocol	P6-protocol	*P* value
No. of cycles	700	700	
Biochemical pregnancy rate, n(%)	350 (50.00%)	400 (57.14%)	0.01
Clinical pregnancy rate, n(%)	309 (44.14%)	353 (50.43%)	0.02
First-trimester abortion rate, n(%)	64 (20.71%)	64 (18.13%)	0.40
Ectopic pregnancy rate, n(%)	6 (1.94%)	8 (2.27%)	0.77
Live birth rate, n(%)	221 (31.57%)	269 (38.43%)	0.01
Newborn sex, n(%)			0.08
Female	90 (40.72%)	131 (48.70%)	
Male	131 (59.28%)	138 (51.30%)	
Gestational age, wk	37.99 ± 1.77	38.13 ± 1.793	0.37
Gestational age category, n(%)			0.22
28-36 wk	31 (14.03%)	28(10.41%)	
≥37 wk	190 (85.97%)	241 (89.59%)	
Birth weigh, g	3,398.78 ± 569.28	3,389.32 ± 557.69	0.85
Birth weight category, n(%)			0.85
Normal (2,500 g-4,000 g)	185 (83.71%)	224 (83.27%)	
Low (<2,500 g)	5 (2.26%)	9 (3.35%)	
Very low(<1,500 g)	3 (1.36%)	5 (1.86%)	
Macrosomia (>4,000 g)	28 (12.67%)	31 (11.52%)	
Neonatal malformation rate^*^, n(%)	2 (0.90%)	2 (0.74%)	0.10

Values are presented as mean ± standard deviation or n (%).Statistically significant P values are reported as P < 0.05.

*Neonatal malformations: two congenital heart disease in P7-protocol; two congenital talipes valgus in P6-protocol. wk, week.

Notably, live birth rate was significantly higher in the P6-group than in the P7-group, with a difference of 6.86% (38.43% *versus* 31.57%, *P* = *0.01*). Likewise, clinical pregnant rate (50.43% *versus* 44.14%, *P = 0.02*) and biochemical pregnancy rate (57.14% *versus* 50.00%, *P* = *0.01*) were significantly higher in the P6-group respectively. However, the rates of first-trimester abortion (18.13% *versus* 20.71%, respectively, P = 0.40) and ectopic pregnancy (2.27% *versus* 1.94%, respectively, P = 0.77) were comparable between the two groups.

Regarding the offspring’s outcome, the average gestational age and birth weight was comparable between the two groups. For newborns, neonatal sex distribution, preterm birth rate and low-birth-weight/very-low-birth-weight rate were similar in two groups. It is a pity that two congenital heart disease and two congenital talipes valgus occurred in P7-protocol and P6-protocol respectively.

### Subgroup Analysis

Main clinical outcomes including LBR, CPR and first-trimester abortion rate were analyzed between subgroups in **Table 3**. Significantly higher LBRs were observed in patients with age under 35, D5 blastocyst and/or high-quality blastocyst transfer, undergoing HRT combined P6-protocol. Interestingly, the first-trimester abortion rate was comparable in each subgroup.

**Table 3:** Clinical outcomes of subgroups in PS-matched population.

Variable	Live birth rate			Clinical pregnant rate			First-trimester abortion rate		
	P7	P6	P value	P7	P6	P value	P7	P6	P value
Age stratification									
-35, n(%)	175 (32.77%)	228 (44.02%)	<0.01	244 (45.69%)	290 (55.98%)	<0.01	48 (19.67%)	44 (15.17%)	0.17
35-38, n(%)	30 (36.14%)	31 (32.63%)	0.62	43 (51.81%)	41 (43.16%)	0.25	11 (25.58%)	9 (21.95%)	0.70
38-, n(%)	16 (19.28%)	10 (11.49%)	0.16	22 (26.51%)	22 (25.29%)	0.87	5 (22.73%)	11 (50.00%)	0.06
Blastocyst days,n (%)									
D5	186 (35.43%)	224 (43.33%)	0.01	254 (48.38%)	278 (53.77%)	0.08	49 (19.29%)	43 (15.47%)	0.24
D6	35 (20.00%)	45 (24.59%)	0.30	55 (31.43%)	75 (40.98%)	0.06	15 (27.27%)	21 (28.00%)	0.93
Blastocyst quality, n(%)									
High-quality	150 (37.78%)	180 (48.52%)	<0.01	202 (50.88%)	222 (59.84%)	0.01	39 (19.31%)	32 (14.41%)	0.18
Poor-quality	71 (23.43%)	89 (27.05%)	0.30	107 (35.31%)	131 (39.82%)	0.24	25 (23.36%)	32 (24.43%)	0.85
Endometrial preparation									
HRT	187 (31.86%)	244 (40.46%)	<0.01	254 (43.27%)	313 (51.91%)	<0.01	49 (19.29%)	55 (17.57%)	0.60
Suppression HRT	34 (30.09%)	25 (25.77%)	0.49	55 (48.67%)	40 (41.24%)	0.28	15 (27.27%)	9 (22.50%)	0.60

Values are presented as mean ± standard deviation or n (%). Statistically significant P values are reported as P < 0.05.

High-quality=both the inner cell mass and trophectoderm scores were above grade B (3BB).

When subgroup analysis was performed by age stratification, significantly higher LBR (44.02% *versus* 32.77%, *P <0.01*) and CPR (55.98% *versus* 45.69%, *P <0.01*) were reported in P6-group among patients below 35 years old. As for patients aged 35–38 and over 38, LBR and CPR were comparable in subgroups.

Subgroup analysis by blastocyst development days had shown that, when D5 blastocyst transferred, significantly higher LBR among patients with P6-protocol (43.33% *versus* 35.43%, *P = 0.01*), but similar CPR (53.77% *versus* 48.38%, *P = 0.08*) were found respectively. Interestingly, when D6 blastocyst transferred, comparable LBR (24.59% *versus* 20.00%, *P = 0.30*) and CPR (40.98% *versus* 31.43%, *P = 0.06*) were reported between groups.

When subgroup analysis was performed by blastocyst quality, significantly higher LBR (48.52% *versus* 37.78% *P* <0.01) and CPR (59.84% *versus* 50.88%, *P* = 0.01), as well as comparable abortion rate (14.41% *versus* 19.31%, *P* = 0.18) were reported in P6-group with high-quality blastocysts transfer. However, when poor-quality blastocysts transferred, similar LBR (27.05% *versus* 23.43% *P* = 0.30) and CPR (39.82% *versus* 35.31%, *P* = 0.24), as well as comparable abortion rate (24.43% *versus* 23.36%, *P* = 0.85) were found between groups.

In addition, subgroup analysis based on endometrial preparation protocol revealed significantly higher LBR (40.46% *versus* 31.86%, *P <0.01*) and higher CPR (51.91% *versus* 43.27%, *P <0.01*) in patients undergoing HRT with P6-protocol. Once suppression HRT used, comparable LBR (25.77% *versus* 30.09%, P = 0.49) and similar CPR (41.24% *versus* 48.67%, P = 0.28) were recorded in this study.

## Discussion

It is known that improper duration of progesterone before transfer will cause synchronize between the endometrium and embryo, meanwhile, delayed implantation will bring early pregnancy loss (17). Several studies have been conducted to explore the question that “What is the optimal duration of progesterone administration before transferring a vitrified-warmed blastocyst”, but no consensus has been reached (12, 13). In view of the trend of single blastocyst transfer, this study focused on optimal duration of progesterone administration before transferring vitrified-warmed blastocyst in HRT cycles.

Firstly, the results of this retrospective cohort study demonstrated that single blastocyst FET with P6-protocol was related to better clinical outcomes comparing with P7-protocol. To our knowledge, this is a study with the largest sample size at present comparing these two progesterone administration protocols with additional detailed subgroup analysis. Notably, this is the largest Chinese study of its kind and has great value considering the trend of single blastocyst transfer recently. This detailed real-world study can provide useful information to help reproductive physicians make clinical strategy. Furthermore, this PS-well-matched study is more conducive to reliable conclusions.

In theory, reproductive physician should transfer a day 5 blastocyst at 5 days after suspected ovulation. However, the optimal day of blastocyst transfer in natural cycle FET has been still debated because of different criteria of ovulation day. It is known that the LH surge precedes ovulation and luteinization, and always begins between midnight and 08:00 in over two-thirds of women, about 34–36 h prior to follicle rupture (18, 19). Serum or urinary LH surge testing and ultrasound monitoring have been reported to dictate transfer timing in natural cycle (20–22). When considering the obstetric complications and potential neonatal outcomes, back to nature cycle means a lot for FET (8, 9, 23).

Just as important, HRT is applicable for patients with an irregular menstrual cycle, ovulation disorder, or poor endometrial and follicular development in NC in our center (14). Because of anovulation and absence of corpus luteum, the progesterone duration before transfer in HRT is crucial to pregnancy outcomes in FET cycles (7, 10). The optimal progesterone duration prior to embryo transfer has remained an elusive topic since the start of FET. Like natural cycle, the timing of blastocyst transfer in HRT is also controversial. Basing on available evidence, the optimal progesterone duration before transfer in HRT is assumed to be equal to the theoretical day of ovulation or 1 day later (7). In HRT cycle, blastocyst was proposed to be transferred on day (embryonic age + 1) of progesterone administration, annotated as P+ embryonic age (7). In our study, we followed the “embryonic age + 1” protocol in P6-protocol and found better clinical outcomes comparing to P7-protocol. Furthermore, P6-protocol is preferred to P7-protocol among most subgroup analysis.

Inconsistent with this study, an increased difference of 16% in clinical pregnancy rate was seen when FET was on the fifth than on the seventh day of progesterone administration in HRT cycle, but the difference was not statistically significant (12). Meanwhile, the retrospective cohort study from the same team found that FET on the sixth day of progesterone administration resulted in similar LBRs to those of embryo transfer on the seventh day of progesterone administration (13). The different conclusions of these studies may be related to the following factors. First, different protocols of endometrial preparation and luteal phase support were used in the retrospective cohort study, those could impact on results. Second, different sets of criteria of planning endometrium thickness also help explain part of the inconsistency. Besides, for retrospective analysis, if case matching is carried out, the conclusion may be different.

Another main finding from the current study was that significantly higher LBR and Clinical pregnant rate have been stated with D5 blastocyst than that with D6 blastocyst (**Supplemental Table S1**). First-trimester abortion rate was similar in subgroups. Previous studies have shown that implantation, clinical pregnant rate and LBR were significantly higher following D5 transfer compared to D6 transfer (14, 24–26). Interestingly, data from our center (from January 2014 to June 2015) stated that high-quality D6 blastocysts in frozen-thawed cycles had similar developmental potential and pregnancy outcomes compared to high-quality D5 blastocysts (14). We hypothesis the following causes lead to differences between these two studies:1) previous study included double blastocyst transfer cycles and that may affect the trend of pregnancy outcomes; and 2) part of the blastocysts in previous study come from the slow-freeze method of cryopreservation and that may affect final results.

In addition, consistent with previous studies, for couples who obtain both D5 and D6 blastocysts after embryo culture, it appears reasonable to transfer high-quality D5 firstly in order to limit time to pregnancy (26). Basing on our data, we suggest the order of priority for frozen-thawed blastocysts transfer cycle was as follows: 1) In HRT cycle, P6-protocol is preferred to P7-protocol; and 2) preferential selection of blastocyst: D5 is preferred to D6, as well as high-quality is preferred poor-quality.

Several limitations exist in the current study. First, this study was of retrospective design; thus, potential bias factors cannot be fully identified and addressed. In order to reduce the influence of confounding factors, we performed a matched propensity score (PSM) analysis to assess covariates. Furthermore, we conducted subgroup analysis as much as possible in **Table 3**, including age stratification analysis (–35, 35–38, 38–), blastocyst days (D5, D6), blastocyst quality (high-quality, low-quality) and endometrial preparation protocols (HRT, suppression HRT). Second, no standardized luteal support scheme was conducted between the two groups, which may have a potential impact on pregnancy outcomes. Third, parts of cases were excluded after PSM, which may lead to bias of this real world study.

In conclusion, this retrospective analysis demonstrated that frozen-thawed blastocyst transfer on the sixth day of progesterone administration is related to higher live birth rate compared with transfer on the seventh day in HRT cycles, especially in patients with age under 35, D5 blastocyst and/or high-quality blastocyst transfer. In the face of embryo selection before transfer, preferential selection of D5 and high-quality blastocyst will shorten time to pregnancy.

## Data Availability Statement

The original contributions presented in the study are included in the article/**Supplementary Material**. Further inquiries can be directed to the corresponding author.

## Ethics Statement

The studies involving human participants were reviewed and approved by Institutional Review Board (IRB) of First Affiliated Hospital of Zhengzhou University. The patients/participants provided their written informed consent to participate in this study.

## Author Contributions

XY and ZB contributed to the conception, design, acquisition and interpretation of data, and drafting of the manuscript. LH supervised the study. All authors contributed to the article and approved the submitted version.

## Conflict of Interest

The authors declare that the research was conducted in the absence of any commercial or financial relationships that could be construed as a potential conflict of interest.

## Publisher’s Note

All claims expressed in this article are solely those of the authors and do not necessarily represent those of their affiliated organizations, or those of the publisher, the editors and the reviewers. Any product that may be evaluated in this article, or claim that may be made by its manufacturer, is not guaranteed or endorsed by the publisher.

## References

[1] Peeraer K Debrock S Laenen A De Loecker P Spiessens C De Neubourg D. The Impact of Legally Restricted Embryo Transfer and Reimbursement Policy on Cumulative Delivery Rate After Treatment With Assisted Reproduction Technology. Hum Reprod (2014) 29(2):267–75. 10.1093/humrep/det405 24282120

[2] Long X Wang Y Wu F Li R Chen L Qian W. Pregnancy Outcomes of Single/Double Blastocysts and Cleavage Embryo Transfers: A Retrospective Cohort Study of 24,422 Frozen-Thawed Cycles. Reprod Sci (2020) 27(12):2271–8. 10.1007/s43032-020-00247-x PMC7593290 32840740

[3] Blockeel C Drakopoulos P Santos-Ribeiro S Polyzos NP Tournaye H. A Fresh Look at the Freeze-All Protocol: A SWOT Analysis. Hum Reprod (2016) 31(3):491–7. 10.1093/humrep/dev339 26724793

[4] Mizrachi Y Horowitz E Farhi J Raziel A Weissman A. Ovarian Stimulation for Freeze-All IVF Cycles: A Systematic Review. Hum Reprod Update (2020) 26(1):118–35. 10.1093/humupd/dmz037 31867625

[5] Practice Committees of the American Society for Reproductive Medicine and the Society for Assisted Reproductive Technology. The Use of Preimplantation Genetic Testing for Aneuploidy (PGT-A): A Committee Opinion. Fertil Steril (2018) 109(3):429–36. 10.1016/j.fertnstert.2018.01.002 29566854

[6] Kuang Y Chen Q Fu Y Wang Y Hong Q Lyu Q. Medroxyprogesterone Acetate is an Effective Oral Alternative for Preventing Premature Luteinizing Hormone Surges in Women Undergoing Controlled Ovarian Hyperstimulation for *In Vitro* Fertilization. Fertil Steril (2015) 104(1):62–70. 10.1016/j.fertnstert.2015.03.022 25956370

[7] Mackens S Santos-Ribeiro S van de Vijver A Racca A Van Landuyt L Tournaye H. Frozen Embryo Transfer: A Review on the Optimal Endometrial Preparation and Timing. Hum Reprod (2017) 32(11):2234–42. 10.1093/humrep/dex285 29025055

[8] Saito K Kuwahara A Ishikawa T Morisaki N Miyado M Miyado K. Endometrial Preparation Methods for Frozen-Thawed Embryo Transfer are Associated With Altered Risks of Hypertensive Disorders of Pregnancy, Placenta Accreta, and Gestational Diabetes Mellitus. Hum Reprod (2019) 34(8):1567–75. 10.1093/humrep/dez079 31299081

[9] Singh B Reschke L Segars J Baker VL. Frozen-Thawed Embryo Transfer: The Potential Importance of the Corpus Luteum in Preventing Obstetrical Complications. Fertil Steril (2020) 113(2):252–7. 10.1016/j.fertnstert.2019.12.007 PMC7380557 32106972

[10] Nawroth F Ludwig M. What is the ‘Ideal’ Duration of Progesterone Supplementation Before the Transfer of Cryopreserved-Thawed Embryos in Estrogen/Progesterone Replacement Protocols? Hum Reprod (2005) 20(5):1127–34. 10.1093/humrep/deh762 15695314

[11] Theodorou E Forman R. Live Birth After Blastocyst Transfer Following Only 2 Days of Progesterone Administration in an Agonadal Oocyte Recipient. Reprod BioMed Online (2012) 25(4):355–7. 10.1016/j.rbmo.2012.06.011 22868081

[12] van de Vijver A Drakopoulos P Polyzos NP Van Landuyt L Mackens S Santos-Ribeiro S. Vitrified-Warmed Blastocyst Transfer on the 5th or 7th Day of Progesterone Supplementation in an Artificial Cycle: A Randomised Controlled Trial. Gynecol Endocrinol (2017) 33(10):783–6. 10.1080/09513590.2017.1318376 28443690

[13] Roelens C Santos-Ribeiro S Becu L Mackens S Van Landuyt L Racca A. Frozen-Thawed Blastocyst Transfer After 6 or 7 Days of Progesterone Administration: Impact on Live Birth Rate in Hormone Replacement Therapy Cycles. Fertil Steril (2020) 114(1):125–32. 10.1016/j.fertnstert.2020.03.017 32553469

[14] Yang H Yang Q Dai S Li G Jin H Yao G. Comparison of Differences in Development Potentials Between Frozen-Thawed D5 and D6 Blastocysts and Their Relationship With Pregnancy Outcomes. J Assist Reprod Genet (2016) 33(7):865–72. 10.1007/s10815-016-0712-6 PMC4930781 27098058

[15] Austin PC. An Introduction to Propensity Score Methods for Reducing the Effects of Confounding in Observational Studies. Multivariate Behav Res (2011) 46(3):399–424. 10.1080/00273171.2011.568786 21818162 PMC3144483

[16] Austin PC. A Tutorial and Case Study in Propensity Score Analysis: An Application to Estimating the Effect of In-Hospital Smoking Cessation Counseling on Mortality. Multivariate Behav Res (2011) 46(1):119–51. 10.1080/00273171.2011.540480 PMC3266945 22287812

[17] Wilcox AJ Baird DD Weinberg CR. Time of Implantation of the Conceptus and Loss of Pregnancy. N Engl J Med (1999) 340(23):1796–9. 10.1056/NEJM199906103402304 10362823

[18] Cahill DJ Wardle PG Harlow CR Hull MG. Onset of the Preovulatory Luteinizing Hormone Surge: Diurnal Timing and Critical Follicular Prerequisites. Fertil Steril (1998) 70(1):56–9. 10.1016/S0015-0282(98)00113-7 9660421

[19] Hoff JD Quigley ME Yen SS. Hormonal Dynamics at Midcycle: A Reevaluation. J Clin Endocrinol Metab (1983) 57(4):792–6. 10.1210/jcem-57-4-792 6411753

[20] Irani M Robles A Gunnala V Reichman D Rosenwaks Z. Optimal Parameters for Determining the LH Surge in Natural Cycle Frozen-Thawed Embryo Transfers. J Ovarian Res (2017) 10(1):70. 10.1186/s13048-017-0367-7 29037231 PMC5644145

[21] Cardellicchio L Reschini M Paffoni A Guarneri C Restelli L Somigliana E. Frozen-Thawed Blastocyst Transfer in Natural Cycle: Feasibility in Everyday Clinical Practice. Arch Gynecol Obstet (2017) 295(6):1509–14. 10.1007/s00404-017-4383-z 28455581

[22] Haouzi D Entezami F Torre A Innocenti C Antoine Y Mauries C. Customized Frozen Embryo Transfer After Identification of the Receptivity Window With a Transcriptomic Approach Improves the Implantation and Live Birth Rates in Patients With Repeated Implantation Failure. Reprod Sci (2021) 28(1):69–78. 10.1007/s43032-020-00252-0 32725589 PMC7782404

[23] Maheshwari A Pandey S Amalraj RE Shetty A Hamilton M Bhattacharya S. Is Frozen Embryo Transfer Better for Mothers and Babies? Can Cumulative Meta-Analysis Provide a Definitive Answer? Hum Reprod Update (2018) 24(1):35–58. 10.1093/humupd/dmx031 29155965

[24] Ferreux L Bourdon M Sallem A Santulli P Barraud-Lange V Le Foll N. Live Birth Rate Following Frozen-Thawed Blastocyst Transfer Is Higher With Blastocysts Expanded on Day 5 Than on Day 6. Hum Reprod (2018) 33(3):390–8. 10.1093/humrep/dey004 29394365

[25] Haas J Meriano J Laskin C Bentov Y Barzilay E Casper RF. Clinical Pregnancy Rate Following Frozen Embryo Transfer Is Higher With Blastocysts Vitrified on Day 5 Than on Day 6. J Assist Reprod Genet (2016) 33(12):1553–7. 10.1007/s10815-016-0818-x PMC5171889 27714479

[26] Bourdon M Pocate-Cheriet K Finet DBA Grzegorczyk-Martin V Amar HA Arbo E. Day 5 Versus Day 6 Blastocyst Transfers: A Systematic Review and Meta-Analysis of Clinical Outcomes. Hum Reprod (2019) 34(10):1948–64. 10.1093/humrep/dez163 PMC7967799 31644803

