# Supplementary material for: Live Birth Rate of Frozen-Thawed Single Blastocyst Transfer After 6 or 7 Days of Progesterone Administration in Hormone Replacement Therapy Cycles: A Propensity Score-Matched Cohort Study
Source: Front Endocrinol (Lausanne). 2021 Aug 11;12:706427. doi: 10.3389/fendo.2021.706427 (PMC8385316; doi:10.3389/fendo.2021.706427)
Supplement: Supplementary file 1 [file Table_1.doc]

|  | P7 | | | P6 | | |
| --- | --- | --- | --- | --- | --- | --- |
| Variable | D5 | P6 | *P* value | D5 | D6 | *P* value |
| Live birth rate, n(%) | 186(35.43%) | 35(20.00%) | ＜0.01 | 224(43.33%) | 45(24.59%) | ＜0.01 |
| Clinical pregnant rate, n(%) | 254(48.38%) | 55(31.43%) | ＜0.01 | 278(53.77%) | 75(40.98%) | ＜0.01 |
| First-trimester abortion rate, n(%) | 49(8.52%) | 21(11.48%) | 0.20 | 49(9.33%) | 15(8.57%) | 0.76 |

Supplemental Table [1](#_bookmark30). Clinical outcomes of D5/D6 subgroups in PS-matched population
